# Phosphorylation of mTOR Ser2481 is a key target limiting the efficacy of rapalogs for treating hepatocellular carcinoma

**DOI:** 10.18632/oncotarget.10161

**Published:** 2016-06-18

**Authors:** Kosuke Watari, Ayumi Nishitani, Tomohiro Shibata, Masaki Noda, Akihiko Kawahara, Jun Akiba, Yuichi Murakami, Hirohisa Yano, Michihiko Kuwano, Mayumi Ono

**Affiliations:** ^1^ Department of Pharmaceutical Oncology, Graduate School of Pharmaceutical Sciences, Kyushu University, Fukuoka, Japan; ^2^ Department of Diagnostic Pathology, Kurume University Hospital, Kurume, Japan; ^3^ Department of Pathology, Kurume University School of Medicine, Kurume, Japan; ^4^ Cancer Translational Research Center, St. Mary's Institute of Health Sciences, Kurume, Japan

**Keywords:** mTOR Ser2481, mTORC1, Raptor, rapalogs, hepatocellular carcinoma

## Abstract

Hepatocellular carcinoma (HCC) is one of the most common cancers worldwide. Although recent studies facilitate the identification of crucial genes and relevant regulatory pathways, therapeutic approaches against advanced HCC are insufficiently effective. Therefore, we aimed here to develop potent therapeutics to provide a reliable biomarker for the therapeutic efficacy in patients with HCC. To this end, we first compared the cytotoxic effects of various anti-cancer drugs between well differentiated (HAK-1A) and poorly differentiated (HAK-1B) cell lines established from a single HCC tumor. Of various drug screened, HAK-1B cells were more sensitive by a factor of 2,000 to the mTORC1 inhibitors (rapalogs), rapamycin and everolimus, than HAK-1A cells. Although rapalogs inhibited phosphorylation of mTOR Ser2448 in HAK-1A and HAK-1B cells, phosphorylation of mTOR Ser2481 was specifically inhibited only in HAK-1B cells. Silencing of Raptor induced apoptosis and inhibited the growth of only HAK-1B cells. Further, three other cell lines established independently from the tumors of three patients with HCC were also approximately 2,000-fold times more sensitive to rapamycin, which correlated closely with the inhibition of mTOR Ser2481 phosphorylation by rapamycin. Treatment with everolimus markedly inhibited the growth of tumors induced by poorly differentiated HAK-1B and KYN-2 cells and phosphorylation of mTOR Ser2481 *in vivo*. To our knowledge, this is the first study showing that the phosphorylation of mTOR Ser2481 is selectively inhibited by rapalogs in mTORC1-addicted HCC cells and may be a potential reliable biomarker for the therapeutic efficacy of rapalogs for treating HCC patients.

## INTRODUCTION

Advanced hepatocellular carcinoma (HCC) is one of the most common cancers worldwide, although there is no effective therapy for patients with advanced HCC [1]. The pathogenesis of HCC comprises numerous genetic and epigenetic changes that accumulate for years [2]. Focusing on abnormal genomic changes that occur in HCC cells during disease progression is an established strategy for the development of new therapies [3, 4]. Activation of various cellular signaling molecules such as the mammalian target of rapamycin (mTOR), epidermal growth factor receptor (EGFR), and insulin-like growth factor receptor (IGFR) may contribute to the malignant progression of HCC [1]. Although systemic chemotherapy is not effective, the multikinase inhibitor sorafenib significantly increases the overall survival of patients with advanced HCC [5, 6]. However, therapeutic effects of sorafenib are limited to selected patients with preserved liver function (Child-Pugh A), and its side effects limit its applicability as well [1].

Expression profiling studies show that the *MYC* oncogene drives the progression of dysplastic nodules to early HCC [7]. Mutations in phosphoinositide-3-kinase (PI3K), catalytic, alpha polypeptide (PIK3CA), TP53, T cell factor 1 (TCF1), and WNT signaling pathway as well as AKT activation predict unfavorable outcomes of patients with HCC [8–11]. However, the contribution of such oncogenic changes to the progression of HCC is unknown.

To identify molecular targets that might determine the aggressive phenotype of HCC, one approach compares biochemical characteristics associated with cell growth, survival, and drug sensitivity between benign and malignant HCC cells *in vitro*. Certain HCCs form single nodule-in-nodule tumors that comprise well differentiated and poorly differentiated cancer cells in the outer and inner nodules, respectively [12, 13]. Therefore, we established well or poorly differentiated HCC cell lines (HAK-1A and HAK-1B, respectively) from a single HCC tumor with a nodule-in-nodule appearance [12]. These HCC cell lines harbor identical structural abnormalities on chromosomes 2 and 7 and an identical point mutation in *TP53* codon 242 [12, 14], indicating that HAK-1A and HAK-1B cells are derived from the same clone. HAK-1B cells express much lower levels of the specific differentiation marker, the N-myc downstream regulated gene 1 (NDRG1), compared with HAK-1A cells [15], indicating the poorly differentiated phenotype of HAK-1B cells. HAK-1B formed tumors in nude mice, but HAK-1A did not [15].

Here we compared the biochemical characteristics of HAK-1A and HAK-1B cells as well as those of other human HCC cell lines. We discovered that AKT was constitutively phosphorylated in HAK-1B cells, which were 2,000-fold more sensitive to the mTORC1 inhibitors rapamycin and everolimus compared with HAK-1A cells. Treatment with everolimus markedly inhibited the growth of tumors induced by poorly differentiated HAK-1B and KYN-2 cells in nude mice as well as phosphorylation of mTOR Ser2481. Our findings indicate that inhibition of mTOR Ser2481 phosphorylation might limit the sensitivity of HCC cells to rapalogs.

## RESULTS

### PI3K/AKT signaling is constitutively activated in HAK-1B cells

**Figure 1:**
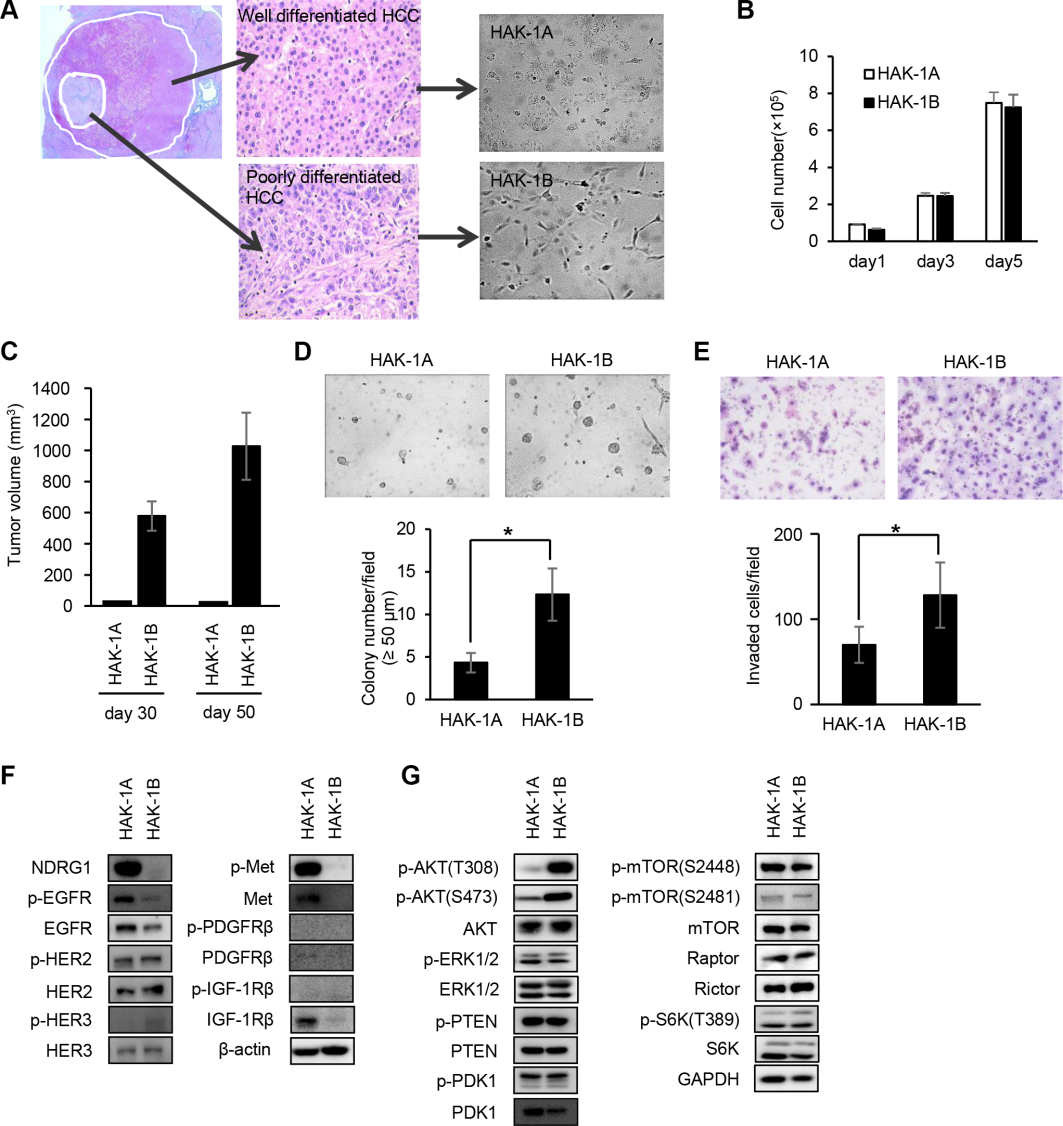
Comparison of the biological and biochemical characteristics of HAK-1A and HAK-1B cells (**A**) Morphology of HCC cell lines in culture. HAK-1A shows cobblestone-like morphology, and HAK-1B shows a fibroblastic morphology when cultured in plastic dishes. A single HCC tumor showing a nodule-in-nodule appearance. The well differentiated HAK-1A and poorly differentiated HAK-1B cell lines were derived from the outer and inner nodules of the same tumor, respectively. (**B**, **C**) Comparison of cell proliferation rates *in vitro* (B), and tumor growth rates on days 30 and 50 in nude mice (C) engrafted with HAK-1A and HAK-1B cells (*n* = 3). Each bar is the average ± standard deviation (SD). (**D**) Comparison of colony formation under “Matrigel on top” culture conditions between HAK-1A and HAK-1B cells. Representative images of colonies of HAK-1A and HAK-1B cells incubated for 5 days (upper panel). The number of colonies > 50 μm (lower panel) (*n* = 3). Each bar is the average ± standard deviation (SD), *P < 0.05 (two-tailed Student *t test*). (original magnification ×40) (**E**) Comparison of invasion of Matrigel between HAK-1A and HAK-1B cells. Representative images of invaded cells incubated for 24 hr (upper panel), and the number of invaded cells (lower panel) (*n* = 3). Each bar is an average ± SD, *P < 0.05 (two-tailed Student *t* test). (original magnification ×40) (**F**) Comparison of expression levels of NDRG1 and growth factor receptors in HAK-1A and HAK-1B. β-actin served as loading control. (**G**) Comparison of the expression of downstream effectors in HAK-1A and HAK-1B cells. GAPDH served as loading control.

HAK-1A cells proliferated as a monolayer with a cobblestone-like arrangement, and HAK-1B cells exhibited a fibroblast-like morphology and proliferated as a monolayer with poor cell-to-cell contact (Figure 1A). Although both cell lines grew at similar rates in culture (Figure 1B), only HAK-1B xenografts formed tumors in nude mice (Figure 1C). HAK-1B cells formed > 50 μm colonies were more abundant than those formed by HAK-1A cells (Figure 1D). Further, the ability of HAK-1B cells to invade Matrigel was approximately 2-fold higher compared with that of HAK-1A cells (Figure 1E).

Consistent with our previous study [15], NDRG1 was expressed at low and high levels in HAK-1B and HAK-1A cells, respectively (Figure 1F). The levels of expression of the phosphorylated and unphosphorylated forms of EGFR family members were similar between HAK-1A and HAK-1B cells, although the expression of the phosphorylated and unphosphorylated forms of c-Met, platelet-derived growth factor receptor (PDGFR)β, and IGF-1Rβ were not detectable in HAK-1B cells (Figure 1F). The levels of phosphorylated AKT (Thr308 and Ser473) were higher in HAK-1B cells compared with those in HAK-1A cells (Figure 1G). In contrast, the levels of unphosphorylated and phosphorylated extracellular signal-regulated kinase (ERK) 1/2, phosphatase and tensin homolog deleted from chromosome 10 (PTEN), and phosphoinositide-dependent kinase-1 (PDK1) were similar in both cell lines (Figure 1G).

### HAK-1B cells are highly sensitive to the cytotoxic effects of mTORC1 inhibitors

**Figure 2:**
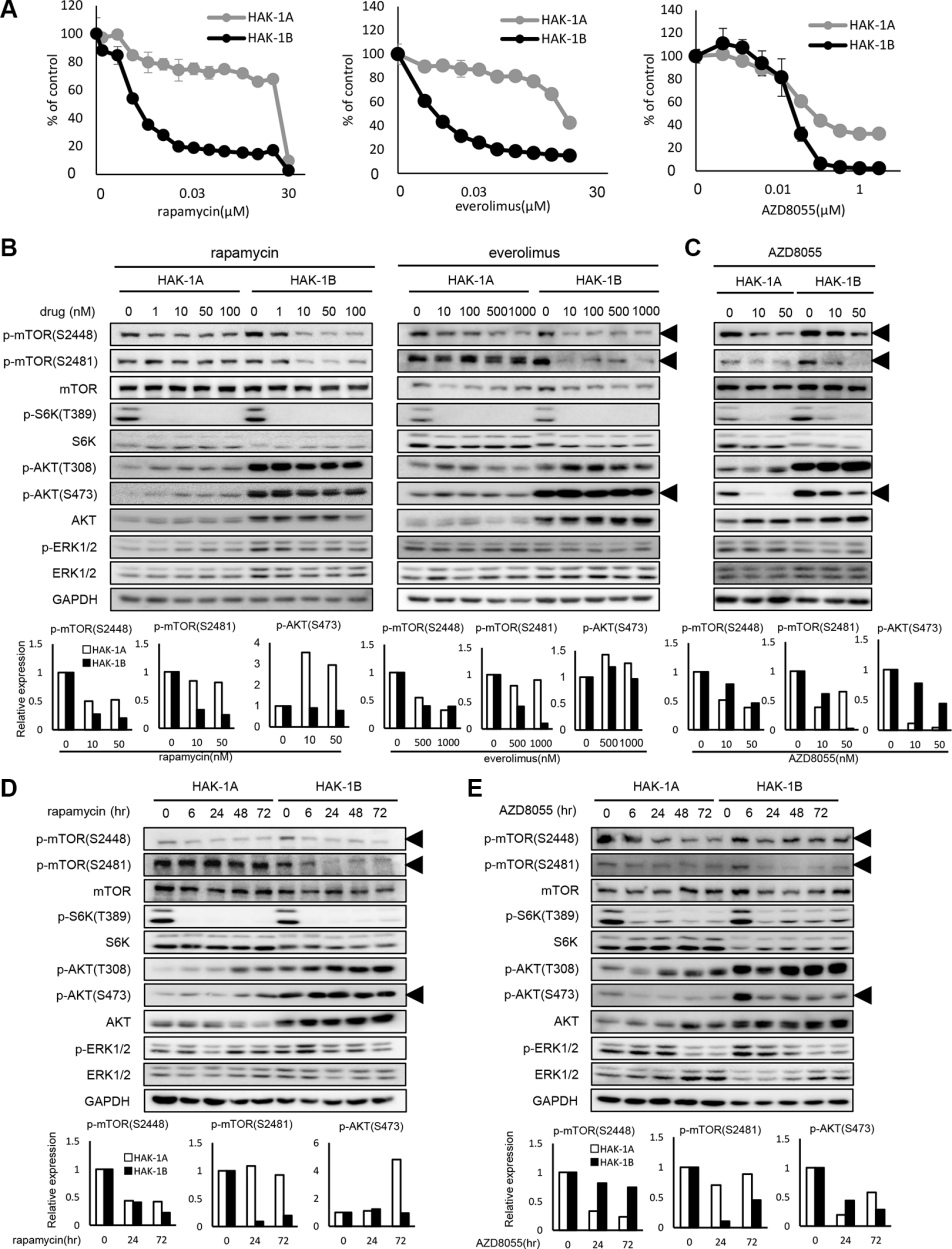
The effect of mTOR inhibitors on the activation of mTOR-related signaling molecules in HAK-1A and HAK-1B cells (**A**) Sensitivity to mTOR inhibitors of HAK-1A and HAK-1B cells. Cells were exposed to various concentrations of the indicated drugs for 72 hr, and drug sensitivity was determined using a WST assay. Each value is the average ± SD of triplicate wells, and each percentage value was calculated by normalizing the raw data to that of the control without drugs (100%). (**B**) The effect of selective mTORC1 inhibitors on the expression and phosphorylation of mTOR and its related signaling molecules. Cells were treated with each inhibitor at the indicated concentrations for 6 hr, and lysates were analyzed using western blotting. The lower panel shows the quantification of expression levels of p-mTOR (Ser2448 and 2481) and p-AKT Ser473 normalized to the value in the absence of drugs. GAPDH served as loading control. (**C**) The effect of 6 hr treatment with AZD8055 on the expression and phosphorylation of mTOR and its related signaling molecules. The lower panel shows the relative expression levels of p-mTOR (Ser2448 and Ser2481) and p-AKT Ser473 normalized to the value in the absence of drugs. GAPDH served as loading control. (**D**) Kinetics of the effect of rapamycin (1 nM) on the expression and phosphorylation of mTOR and its related signaling molecules. In the lower panel, expression levels of p-mTOR (S2448 and S2481) and p-AKT Ser473 are presented as their values normalized to the value at time 0. GAPDH served as loading control. (**E**) Kinetics of the effect of AZD8055 (20 nM) on the expression and phosphorylation of mTOR and its related signaling molecules. In the lower panel, expression levels of p-mTOR (Ser2448 and Ser2481) and p-AKT Ser473 were normalized to the value at time 0. GAPDH served as loading control.

We assessed the sensitivities of HAK-1A and HAK-1B cells to the cytotoxic effects of the multikinase inhibitor sorafenib as well as those of other drugs that target signaling molecules (Table 1) [5]. For example, the cytotoxicity of sorafenib, SU11274 (an inhibitor of c-Met), and picropodophyllin (PPP) (an inhibitor of IGF-1R) was similar for both cell lines (Table 1). Gefitinib and erlotinib (inhibitors of EGFR tyrosine kinase) as well as LY294002 (an inhibitor of PI3K) were 3–5-fold more cytotoxic to HAK-1B cells. In contrast, we found that rapamycin and everolimus were 2,000-fold more cytotoxic to HAK-1B cells. The dual inhibitors of mTORC1/C2, AZD8055 and NVP-BEZ235, were 3–5-fold more cytotoxic for HAK-1B cells (Figure 2A, Table 1).

**Table 1:** Comparison of drug sensitivities of HAK-1A and HAK-1B cells

Drugs	Targets	IC_50_, μM (Relative drug sensitivity)	
		HAK-1A	HAK-1B
sorafenib	RAF1, B-RAF, VEGFR2, PDGFRβ, FLT-3, KIT	2.15 ± 0.87 (1)	3.67 ± 0.09 (1.71)
gefitinib	EGFR	7.87 ± 0.77 (1)	3.07 ± 0.013 (0.39)
erlotinib	EGFR	13.3 ± 1.26 (1)	2.83 ± 0.11 (0.21)
afatinib	EGFR, HER2, HER4	1.76 ± 0.057 (1)	0.376 ± 0.33 (0.21)
SU11274	MET	> 30 (1)	> 30 (1)
PPP	IGF1R	0.278 ± 0.020 (1)	0.322 ± 0.030 (1.16)
PD173074	FGFR1, 3, VEGFR2	7.40 ± 0.57 (1)	5.63 ± 0.17 (0.76)
LY2157299	TGF-βR I	> 100 (1)	> 100 (1)
dasatinib	SRC, ABL, KIT, EPH	1.03 ± 0.36 (1)	2.74 ± 0.73 (2.66)
LY294002	PI3K	18.59 ± 5.55 (1)	3.97 ± 0.21 (0.21)
U0126	MEK1/2	> 30 (1)	4.15 ± 0.79 (< 0.14)
PD98059	MEK1	> 100 (1)	39.01 ± 3.37 (< 0.39)
rapamycin	mTORC1	> 10 (1)	0.0005 ± 0.0000 (< 0.00005)
everolimus	mTORC1	7.11 ± 0.50 (1)	0.0033 ± 0.0002 (0.0005)
AZD8055	mTORC1,C2	0.0816 ± 0.0080 (1)	0.0269 ± 0.0080 (0.33)
NVP-BEZ235	PI3K, mTORC1, C2	0.0156 ± 0.0015 (1)	0.0035 ± 0.0007 (0.22)

IC_50_ values (μM) are presented as average values of triplicate experiments. The relative drug sensitivity for each drug is presented as the IC_50_ of HAK-1B normalized to the IC_50_ of HAK-1A cells.

### Inhibitors of mTORC1 selectively inhibit phosphorylation of mTOR Ser2481

We compared the effects of selective inhibitors of mTORC1 or dual inhibitors of mTORC1/C2 on the phosphorylation of mTOR and related molecules in HAK-1A and HAK-1B cells. The mTORC1-selective inhibitors rapamycin and everolimus decreased the phosphorylation of mTOR Ser2448 and S6K in both cell lines in a dose-dependent manner (Figure 2B), although these inhibitors suppressed phosphorylation of mTOR at Ser2481 only in HAK-1B cells. AKT residues Thr308 and Ser473 were constitutively phosphorylated in HAK-1B cells even in the presence of rapalogs. In contrast, treatment with AZD8055 inhibited the phosphorylation of mTOR Ser2448 and Ser2481 as well as AKT Ser473 in HAK-1A and HAK-1B cells in a dose-dependent manner (Figure 2C). Further, inhibition of the phosphorylation of mTOR Ser2481 by rapamycin varied with time only in HAK-1B cells (Figure 2D). The phosphorylation of AKT residues Thr308 and Ser473 in cells treated with rapamycin increased with time only in HAK-1A cells (Figure 2D). AZD8055 inhibited over time the phosphorylation of mTOR residues Ser2448 and Ser2481 as well as AKT Ser473 in both cell lines (Figure 2E).

### The growth and survival of HAK-1B cells requires Raptor

**Figure 3:**
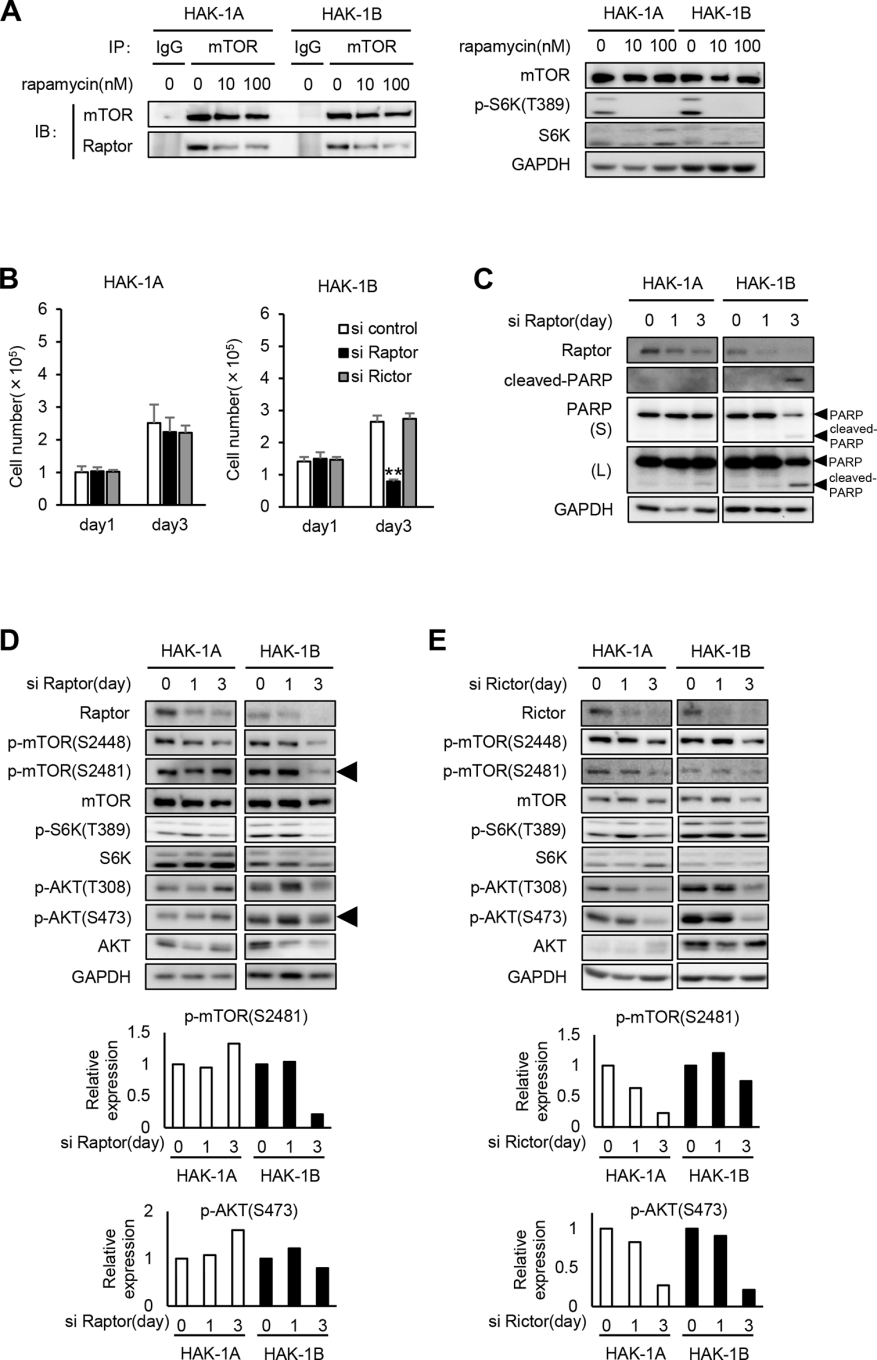
The effect of knockdown of Raptor or Rictor on mTOR and AKT phosphrylation in HAK-1A and HAK-1B cells (**A**) Association of Raptor with mTOR in the absence or presence of rapamycin. Cells were treated with rapamycin at the indicated concentrations for 6 hr, and lysates were analyzed using an immunoprecipitation assay. The immunoprecipitates were subjected to western blot analysis using the indicated antibodies against mTOR or Raptor. GAPDH served as loading control. (**B**) The effect of Raptor or Rictor knockdown on cell growth. Cells were incubated with siRNAs for 1 and 3 days. Each bar is an average ± SD of triplicate wells, **P < 0.01 (two-tailed Student *t* test). (**C**) The effect of Raptor knockdown on the expression of cleaved-PARP. Cells were incubated with siRNA for ≤ 3 days. GAPDH served as loading control. (**D**, **E**) The effect of Raptor (D) or Rictor (E) knockdown on the phosphorylation of mTOR, S6K, and AKT. Cells were incubated with siRNA for 1 and 3 days. In the lower panels, the expression levels of p-mTOR (Ser2481) and p-AKT (Ser473) in siRNAs-treated HAK-1A and HAK-1B cells are normalized to their values on day 0. GAPDH served as loading control.

Rapamycin inhibits mTORC1 activity by dissociating Raptor from mTOR, which prevents access of mTOR to its substrates [16]. Immunoprecipitation-western blot analysis revealed that rapamycin inhibited mTOR binding to Raptor in both cell lines when S6K phosphorylation was inhibited by 10 nM and 100 nM rapamycin (Figure 3A). Rapamycin similarly affected the dissociation of the mTORC1 complex in both cell lines. Moreover, we did not detect mutations in the rapamycin binding and activation domains of mTOR and Raptor in both cell lines, respectively (data was not shown).

We next determined whether Raptor or Rictor, the latter a component of mTORC2, differentially affected cell survival and PI3K/AKT signaling in the two cell lines. The growth of HAK-1B but not HAK-1A cells was selectively inhibited after transfection with a *Raptor*-small interfering RNAs (siRNA) for 3 days (Figure 3B). In contrast, transfection of either cell line for 3 days with a *Rictor*-siRNA did not inhibit the growth of either cell line (Figure 3B). The *Raptor*-siRNA induced apoptosis of only HAK-1B cells (Figure 3C). Moreover, Raptor knockdown markedly inhibited the phosphorylation of mTOR Ser2481 without affecting AKT Ser 473 only in HAK-1B cells (Figure 3D). In contrast, Rictor knockdown suppressed the phosphorylation of mTOR residues Ser2448 and Ser2481 as well as AKT Ser473 to similar levels in both cell lines (Figure 3E).

### Phosphorylation of mTOR Ser2481 is selectively inhibited by rapamycin in other rapamycin-sensitive human HCC cell lines

**Figure 4:**
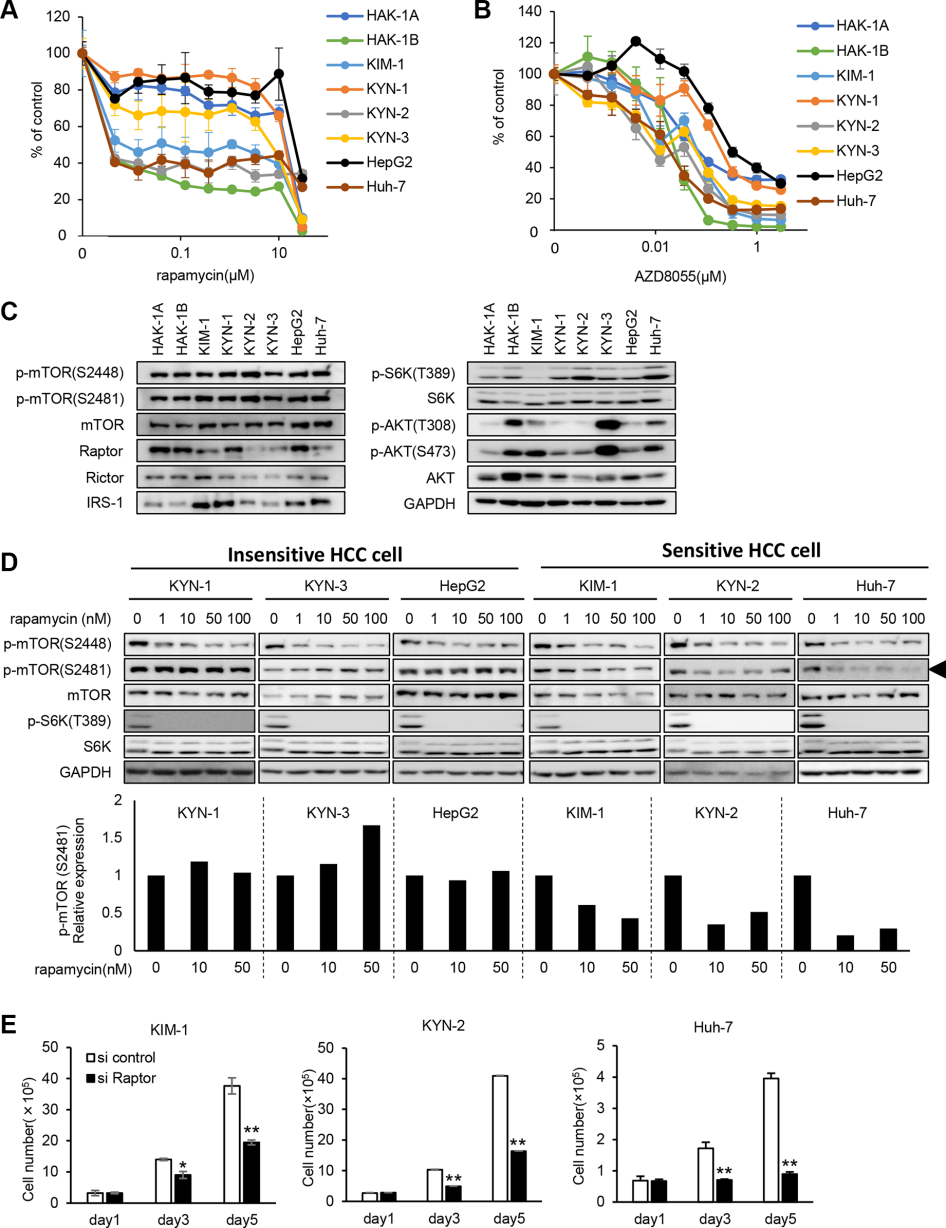
Hypersensitivity to rapamycin and mTORC1-addicted growth of other human HCC cell lines (**A**, **B**) The sensitivities of eight human HCC cell lines to rapamycin (A) and AZD8055 (B) were assessed using a WST assay. Each bar is the average ± SD of triplicate wells. (**C**) Comparison of the expression and phosphorylation of mTOR and its related signaling molecules among eight human HCC cell lines, including HAK-1A and HAK-1B cells. GAPDH served as loading control. (**D**) The effect of rapamycin on the phosphorylation of mTOR and S6K in six cell lines. Cells were treated with rapamycin at indicated concentration for 6 hr, and lysates were analyzed using western blotting. In the lower panel, expression levels of p-mTOR (Ser2481) are normalized to the values in the absence of drugs. GAPDH served as loading control. (**E**) The effect of Raptor knockdown on cell growth. Cells were incubated with a *Raptor*-siRNA for < 5 days. Each bar is the average ± SD of triplicate wells, *P < 0.05; **P < 0.01 (two-tailed Student *t* test).

Three HCC cell lines designated KIM-1, KYN-2, and Huh-7, derived from independent tumors of different patients with HCC, were more sensitive to rapamycin by a factor of 2,000 compared with the KYN-1, KYN-3, and HepG2 cells (Figure 4A). In contrast, the six HCC cell lines showed approximately 10-fold differences in their sensitivities to AZD8055 (Figure 4B). The expression levels of mTOR, phosphorylated (p)-mTOR, mTOR pathway-related molecules, and p-AKT varied among all eight HCC cell lines (Figure 4C).

We assessed previously reported mechanisms that account for the relatively higher sensitivities to mTORC1 inhibitors of the eight HCC cell lines [17–20]. TSC-1 and -2 are negative regulators of mTORC1, and knockdown of their expression induces constitutive activation of the mTORC1 pathway [17]. Further, enhanced p27 expression correlates closely with sensitivity to mTORC1 inhibitors [18]. Expression of TSC-1 and TSC-2 was decreased in KYN-2 cells, and p27 expression was increased in KIM-1 and HAK-1B cells (Supplementary Figure S1A and S1B).

Rapamycin inhibited the phosphorylation of mTOR Ser2448 and S6K in the six HCC cell lines (Figure 4D). Rapamycin selectively inhibited the phosphorylation of mTOR Ser2481 in KIM-1, KYN-2, and Huh-7 cells only (Figure 4D). Treatment with *Raptor*-siRNA suppressed the growth of KIM-1, KYN-2, and Huh-7 cells ≥ 50% of that of the control cells (Figure 4E).

### Tumors formed in nude mice by HAK-1B and KYN-2 cells are highly sensitive to everolimus

**Figure 5:**
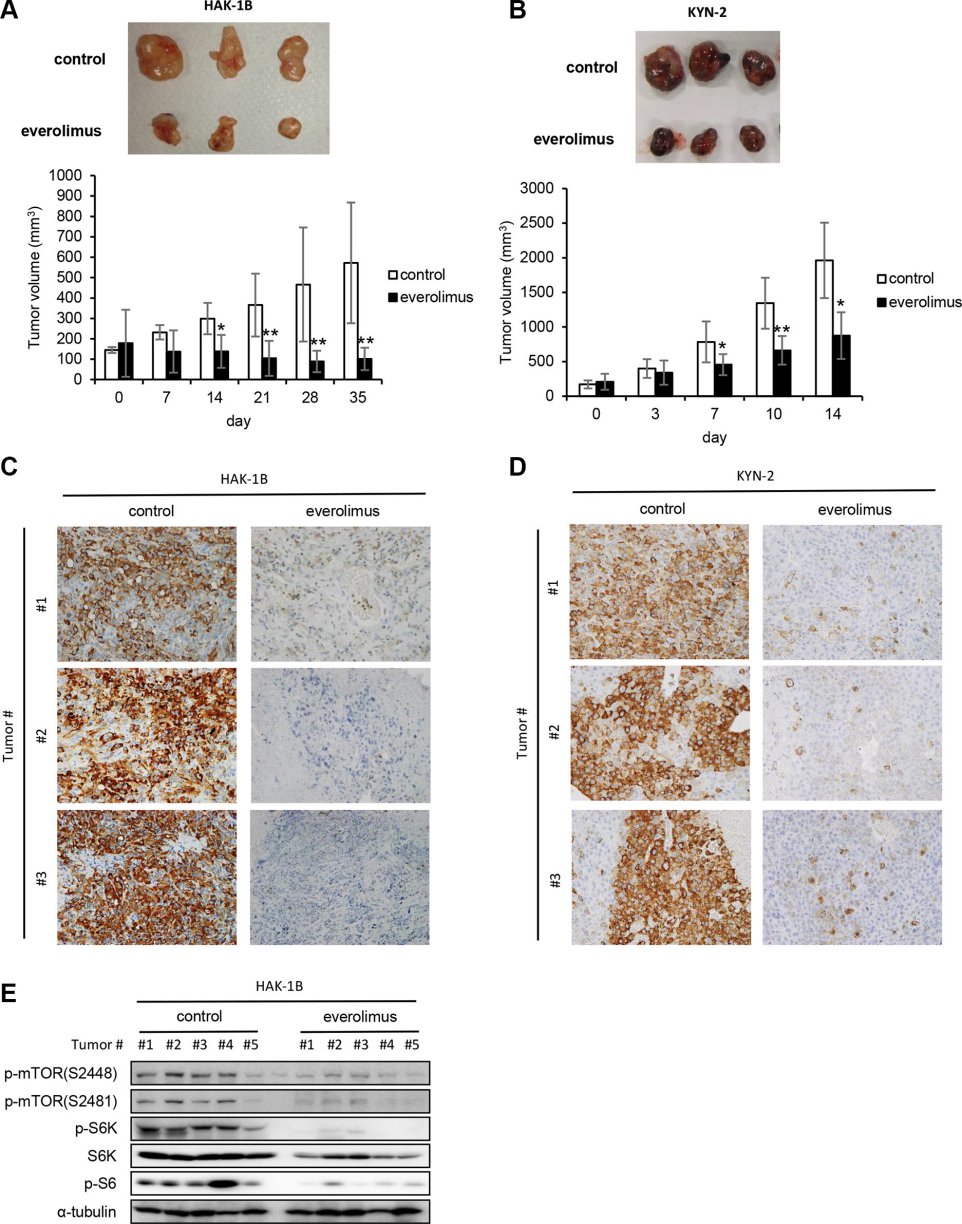
Effect of everolimus on tumor growth and activation of mTOR-related signaling molecules *in vivo* (**A**, **B**) Antitumor effects of everolimus on the growth of HAK-1B (A) and KYN-2 (B) xenografts. Mice were inoculated subcutaneously with HCC cells, and mice with tumors >100 mm^3^ were treated orally with everolimus (2 mg/kg/day) or a control CMC daily from day 0 until days 35 or 14 (*n* = 5 mice per each group). Each bar is the average ± SD, *P < 0.05; **P < 0.01 (two-tailed Student *t* test). (**C**, **D**) Effects of everolimus on S6 phosphorylation in tumors formed by HAK-1B (C) or KYN-2 (D) cells analyzed on days 35 or 14 using immunohistochemistry with an anti-p-S6 antibody. Three tumors of the control and treated groups are shown. (original magnification ×100) (**E**) Inhibitory effects of everolimus on the activation of mTOR-related signaling molecules in tumors. Western blot analysis of the expression of phosphorylated mTOR, S6K, and S6 in five tumors treated with or without everolimus *in vivo*. α-tubulin served as loading control.

KYN-2 cells did not express TSC-2 (Supplementary Figure S1A), suggesting that the higher sensitivity of this cell line to rapamycin was attributable to activation of mTORC1. Therefore, we next asked whether an mTORC1 inhibitor suppressed the formation of tumors formed by HAK-1B or KYN-2 cells *in vivo* and found that HAK-1B and KYN-2 but not HAK-1A cells formed tumors in nude mice (Figure 1C). Treatment with everolimus significantly inhibited the growth of tumors formed by HAK-1B cells (Figure 5A), and reduced the growth of tumors formed by KYN-2 cells (Figure 5B). Immunohistochemical analysis showed markedly decreased expression of phosphorylated S6 in the tumors formed by HAK-1B or KYN-2 cells when the mice were treated with everolimus (Figure 5C and 5D). Western blot analysis of HAK-1B-induced tumors showed marked reduction in the phosphorylation of mTOR residues Ser2448 and Ser2481 as well as S6K and S6 when mice were treated with everolimus (Figure 5E).

## DISCUSSION

**Figure 6:**
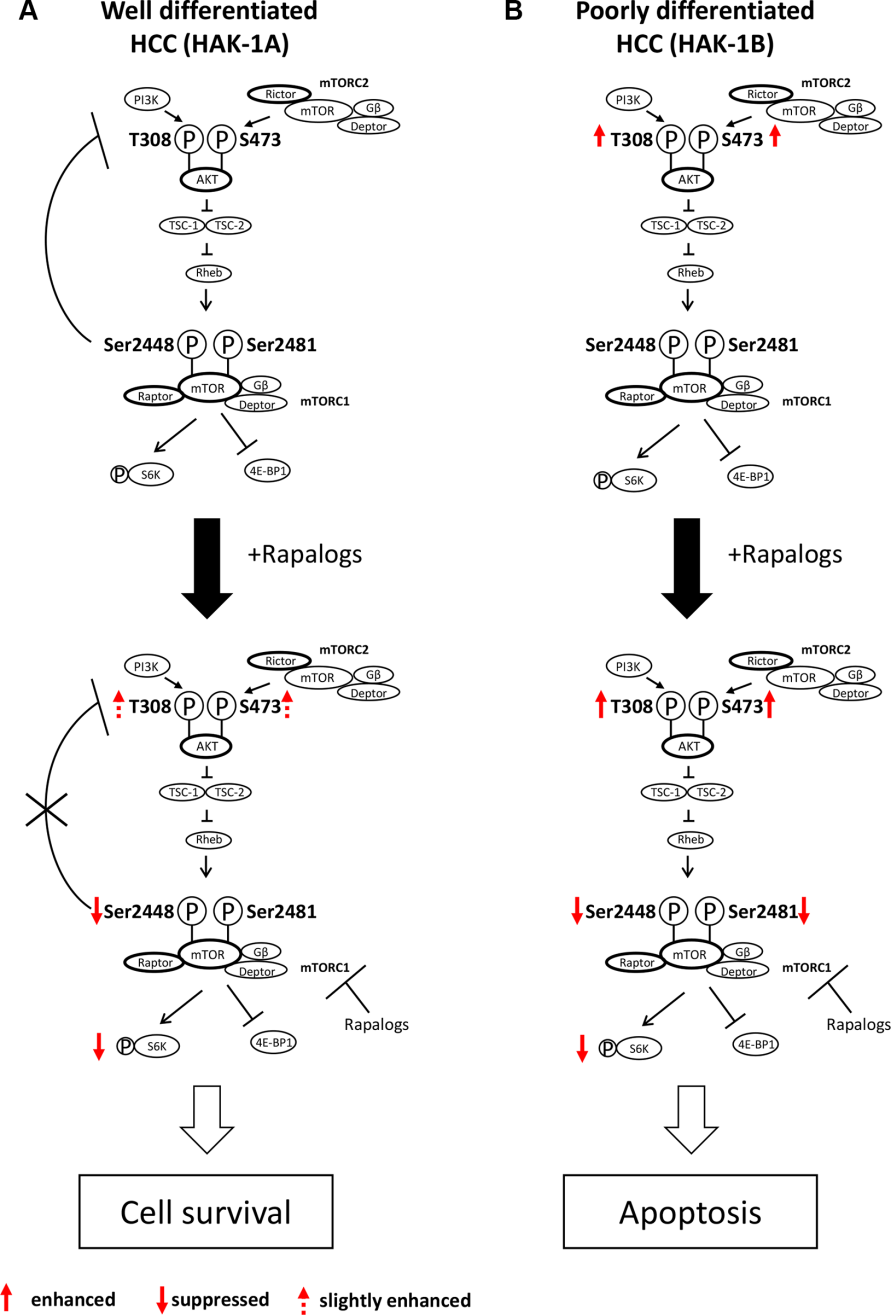
Hypothetical model of the acquisition of hypersensitivity to rapalogs by HCC cells (**A**) Normally, PI3K/AKT activation is under feedback regulation by mTORC1/S6K, such as in well differentiated HCC cells (e.g. HAK-1A cells). Rapalogs inhibit feedback suppression by mTORC1/S6K, possibly through drug-induced inhibition of the phosphorylation of mTOR Ser2448, which is accompanied by PI3K/AKT activation. Rapalogs do not affect the phosphorylation mTOR Ser2481 (mTORC1 complex), and the cells survive in the presence of drugs. (**B**) In contrast, PI3K/AKT is constitutively activated in poorly differentiated HCC cells (e.g. HAK-1B cells), and feedback control of PI3K/AKT by mTORC1 is dysregulated in this cell line. Rapalogs block the phosphorylation of both mTORC1 Ser2448 and Ser2481 that induces the death of HAK-1B cells through suppression of mTORC1-dependent cell growth, survival, and metabolism.

We show here that phosphorylation of mTOR Ser2481 predicts the therapeutic efficacy of mTORC1 inhibitors against HCC. Negative feedback control of PI3K/AKT via mTORC1/S6K in HAK-1A cells is strictly regulated, which is consistent with the results of studies on other cancer and nontumorigenic cell lines [21]. This negative feedback control is blocked by rapalogs when mTOR Ser2448 phosphorylation is inhibited, suggesting a key role for the phosphorylation of mTOR Ser2448 in negative feedback control (Figure 6A). However, rapalogs did not inhibit cell survival or growth signaling in HAK-1A cells. We conclude therefore that the phosphorylation of mTOR Ser2448 was required for the feedback suppression of PI3K/AKT signaling, although it was insufficient to inhibit mTORC1-dependent cell growth and survival signaling. In contrast, rapalogs did not affect the phosphorylation of AKT, suggesting that mTORC1 is independent of the upstream components of the PI3K/AKT pathway in HAK-1B cells (Figure 6B). Phosphorylation of mTOR residues Ser2448 and Ser2481 was inhibited by rapalogs in HAK-1B cells, causing marked suppression of cell survival. Rapalog-induced inhibition of phosphorylation of mTOR residues Ser2448 and Ser2481 is therefore required to inhibit mTORC1/Raptor-addicted cell growth and survival that was independent of AKT activation (Figure 6B).

Similar to HAK-1B cells, the HCC cell lines KIM-1, KYN-2 and Huh-7 were highly sensitive to rapalogs (Figure 4). Phosphorylation of mTOR Ser2481 was blocked by rapamycin only in these three cell lines, whereas the phosphorylation of mTOR Ser2448 was inhibited by rapamycin in rapamycin-sensitive and -insensitive cell lines (Figure 4D). Further, knockdown of Raptor suppressed the growth of the rapamycin-sensitive cell lines (Figure 4E), suggesting that their growth and survival depended on cell signaling through the mTORC1 pathway.

The mechanism of selective inhibition of the phosphorylation of mTOR S2481 by rapalogs and Raptor knockdown is shared by HAK-1B and other rapalog-sensitive HCC cell lines as well as other changes that activate the mTORC1 signaling pathway. For example, the loss of TSC-2 predicts the response to an mTORC1 inhibitor [20], and mTORC1 inhibits p27 expression in close association with sensitivity to mTORC1 inhibitors [18]. However, it is unclear whether such changes of TSC-2 or p27 expression or both are involved in hypersensitivity to rapalogs of these HCC cell lines (Supplementary Figure S1).

The mTOR signaling pathway is upregulated in 15–50% of patients with HCC [22, 23], and mutations of *TSC-1/TSC-2* and *LKB1*, which encode regulators of mTOR signaling, are observed in 5% of HCC patients [24]. In HCC cells, the PI3K/AKT/mTOR pathway is regulated in close association with its upstream and downstream signaling factors, including members of the IRS family of adaptor molecules. Insulin receptor substrate-1 (IRS-1), which is a key downstream regulator of the IR and IGF1R signaling pathways, mediates PI3K/AKT activation via mTORC1-dependent feedback regulation [21]. However, IRS-1 knockdown does not inhibit cell growth or alter sensitivity to rapalogs in the HAK-1B and HAK-1A cell lines (unpublished data), suggesting that it is less likely that IRS-1 is directly involved in the hypersensitivity of HAK-1B cells to rapalogs.

Nowadays, cancer stem cells (CSCs) contribute to tumor resistance to chemotherapy/radiotherapy as well as rapid tumor growth and the epithelial–mesenchymal transition (EMT). Keratin 19 (K19) is known as a biomarker of hepatic progenitor cells (HPCs) that are often found in HCC patients with poor prognosis [25]. Especially, K19 is reported as a key player in tumor invasion in HCC. Therefore, we examined the expression of K19 in HAK-1A and HAK-1B cells (Supplementary Figure S2). Immunohistochemical analysis showed that the expression of K19 was observed only in HAK-1B cells, but not in HAK-1A. Our data supported that HAK-1B exhibited malignant features including higher invasive activity. In addition, Gorvaere et al. recently reported that Laminin-332 induced K19 expression via mTORC2 signaling pathway, and Laminin-332 sustained chemoresistance including sorafenib of HCC cell line [26]. However, in our present study, the cytotoxicity of sorafenib or other drugs except for rapalogs was similar for both HAK-1A and HAK-1B cell lines (Table 1). Based on our data, we concluded that K19 expression might be contributed to higher invasive activity in HAK-1B. Further studies are required to determine whether K19 expression directly alters the sensitivity to mTORC1 inhibitor.

Inhibitors that target mTOR suppress the formation of tumors by HCC cells in experimental model systems [27, 28]. For example, everolimus inhibits tumor growth and increases the survival rate of mice in an HCC xenograft model, and a *Raptor*-specific siRNA reduces the viability of HCC cells *in vitro* [29]. Rapamycin, everolimus, AZD8055, and other mTOR inhibitors show some therapeutic effectiveness when administered to patients with advanced HCC [30, 31]. However, the EVOLVE-1 clinical trial that included 546 randomized patients reported that everolimus did not improve overall survival in patients with advanced HCC whose disease progressed during or after receiving sorafenib or who are intolerant to sorafenib [32]. Many other clinical trials of mTOR-targeting drugs with or without sorafenib are in progress [31].

Serious problems associated with mTOR-targeted therapeutics include the validation of the responses of tumors and how therapy can be optimized for patients with HCC [31]. Although biomarkers that indicate the efficacy of mTOR inhibitors were validated in preclinical and clinical studies of various tumor types, for example, according to *PIK3CA* mutations, PTEN inactivation, and expression levels of p-S6, p-S6K, S6K, and p-AKT [33–35]; the validity of these markers was not confirmed in clinical trials, including those of patients with HCC [31].

The present study is the first to our knowledge to demonstrate that phosphorylation of mTOR Ser2481 was specifically inhibited in HCC cell lines harboring activated mTORC1/Raptor when treated with nanomolar concentrations of mTORC1 inhibitors. We conclude therefore that the phosphorylation of mTOR Ser2481 might provide a reliable marker for the therapeutic efficacy of mTORC1 inhibitors for treating patients with HCC.

In conclusion, we reveal here a novel mechanism involving the phosphorylation of mTOR Ser2481 that is selectively inhibited by rapalogs in mTORC1-addicted HCC cells. Further studies are required to determine how phosphorylation of mTOR Ser2481 drives the growth, survival, and metabolism of HCC cells and why the phosphorylation of mTOR Ser2481 is the key to limiting the sensitivity of HCC cells to rapalogs.

## MATERIALS AND METHODS

### Cell culture

The human HCC cell lines, HAK-1A (well differentiated HCC cell), HAK-1B (poorly differentiated HCC cell), KIM-1 (moderately-differentiated HCC cell), KYN-1 (moderately differentiated HCC cell), KYN-2 (moderately to poorly differentiated HCC cell) and KYN-3 (moderately to poorly differentiated HCC cell) were established at Kurume University from 1984 to 1993 [12, 36–40]. These cells were obtained from Dr. Hirohisa Yano at Kurume University in 2010. HAK-1A and HAK-1B has been established from a single nodule showing a three-layered structure with a different histological grade in each layer from a 55-year-old Japanese male patient with HCC [12]. KIM-1 has been established from a 76-year-old Japanese male patient with HCC [36]. The resected tissue was morphologically proliferative with trabecular pattern corresponding to Edmondson-Steiner's grade II. KYN-1 has been established from a resected HCC of a 58-year-old Japanese male patient with HCC [37]. Original resected HCC was moderately differentiated and proliferated in a solid pattern with vague trabecular structure in part. KYN-2 has been established from a surgical specimen obtained from a 52-year-old Japanese male HCC patient [38]. The originally resected HCC was classified as pleomorphic HCC corresponding to Edmondson-Steiner's grade III with a thick trabecular to solid arrangement. KYN-3 has been established from a 60-year-old Japanese male at autopsy [39]. In the liver section, tumor was proliferative with trabecular pattern corresponding to Edmondson-Steiner's grade II-III. HepG2 cell line (well differentiated HCC cell) was purchased from Riken (Saitama, Japan). Huh-7 (moderately differentiated HCC cell) was purchased from ATCC. All cell lines were passaged for no longer than 6 months. All cell lines were not further tested or authenticated by the authors. These HCC cell lines were maintained in Dulbecco's modified Eagle medium (DMEM) supplemented with 5% or 10% fetal bovine serum (FBS) and incubated in a humidified atmosphere of 5% CO_2_ at 37°C [41, 42].

### Reagents

Anti-NDRG1 antibody was generated as previously described [43]. Other antibodies were purchased as follows: anti-α-tubulin antibody was from Sigma-aldrich Co; anti-EGFR, anti-phospho-EGFR (Tyr1068), anti-phospho-HER3, anti-Met, anti-phospho-Met, anti-PDGFRβ, anti-phospho-PDGFRβ, anti- IGF-1Rβ, anti-phospho-IGF-1Rβ, anti-ERK1/2, anti-phospho-ERK1/2, anti-AKT, anti-phospho-AKT (Thr308 and Ser473), anti-mTOR, anti-phospho-mTOR (Ser2448 and Ser2481), anti-Raptor, anti-Rictor, anti-S6K, anti-phospho-S6K(Thr389), anti-phospho-S6, anti-PTEN, anti-phospho-PTEN, anti-IRS-1, anti-PDK1, anti-PARP, anti-phospho-PDK1, anti-TSC-1, anti-TSC-2, and anti-phospho-4EBP-1 antibodies were from Cell Signaling Technology (Beverly, MA); anti-HER2 and anti-HER3 antibodies were from Santa Cruz Biotechnology (Santa Cruz, CA); anti-glyceraldehyde-3-phosphate dehydrogenase (GAPDH) antibody was from Trevigen Inc. (Gaithersburg, MD): anti-p27 antibody was from BD Biosciences (San Jose, CA): anti-cleaved PARP antibody was from Promega (Madison, WI): anti-phospho-HER2 antibody was from Millipore (Billerica, MA): anti-β-actin was from Abcam (Cambridge, UK).

### Cell growth

HAK-1A and HAK-1B cells (8 × 10^4^) were seeded in 35 mm dishes, and the cell numbers in each dish was counted by a Z2 Coulter Particle Count and Size Analyzer (Beckman Coulter Inc., CA) at 1 or 3 or 5 days [41, 42]. Results were expressed as the mean ± SD of triplicate wells. For cell growth under Raptor or Rictor knockdown condition, HAK-1A, HAK-1B, KIM-1, KYN-2 and Huh-7 cells (1 × 10^5^ cells) were plated in 35 mm dish and following day, cells were transfected with each siRNA. The cell numbers in each dish was counted by a Z2 Coulter Particle Count and Size Analyzer (Beckman Coulter Inc., CA) at 1 or 3 or 5 days. Triplicate dishes were tested at each day. Results were expressed as the mean ± SD of triplicate dishes.

### Ethics statement

All animal experiments were approved by the Ethics of Animal Experiments Committee at Kyushu University Graduate School of Medical Sciences. 6–7-week-old male BALB/c nu/nu athymic nude mice were purchased from CLEA (Saga, Japan), and housed in microisolator cages maintained under a 12 hr light/dark cycle. Water and food were supplied ad libitum. Animals were observed for signs of tumor growth, activity, feeding, and pain in accordance with the guidelines of the Harvard Medical Area Standing Committee on Animals, and accordance with NIH guidelines.

### Xenograft mouse experiment

HAK-1B and KYN-2 cells were suspended in sterile phosphate buffered saline (PBS) at a concentration of 2 × 10^8^ cells/mL, and 100 μL was injected subcutaneously into the right flank of 6–7-week-old male BALB/c athymic nude mice, and the tumor diameters were measured every 3 days from Day 7. Everolimus was dissolved in carboxymethyl cellulose sodium salt (CMC; Wako Pure Chemical Industries, Ltd., Osaka, Japan). Either everolimus (2.0 mg/kg/day) or CMC was administered orally daily. When tumor volume was in the range of 100 – 200 mm^3^, animals were randomly assigned to treatment groups. These mice with tumors larger than 100 mm^3^ was treated with everolimus or CMC. Tumor samples were stored at −80°C for protein analysis or fixed immediately in 10% paraformaldehyde overnight at 4°C and then processed for further histological analysis. The tumor size was measured in two directions using calipers, and the tumor volume (mm^3^) was estimated using the equation: length × (width)^2^ × 0.5 as described previously [44].

### Immunohistochemical analysis

Tissue sections were immunohistochemically stained with anti-phospho-S6 antibody and labeled using the peroxidase method (Histofine SABPO Kit; Nichirei, Tokyo, Japan). Bright-field images were performed with KEYENCE BZ-8000.

### Matrigel on top culture (3-dimensional-growth condition)

For the Matrigel on-top culture in 24 well plates, under the bottom of the well was coated with 100 μl/well of Matrigel (BD Biosciences) and incubated at 37°C for 30 min to allow the gel to solidify. Subsequently, HAK-1A and HAK-1B cells (1 × 10^4^ cells) were resuspended in 1 mL of culture medium (DMEM medium supplemented with 5% FBS), containing 2% Matrigel, and added to the Matrigel-coated well.

### Matrigel invasion assay

BD BioCoat Matrigel Invasion Chambers (BD Bioscience, Bedford, MA) were used according to the manufacturer's instructions. HAK-1A and HAK-1B cells at 7.5 × 10^4^ in serum-free DMEM were seeded onto Matrigel-coated filters in the upper chambers. In the lower chambers, DMEM containing 5% FBS was added as a chemoattractant. After 24 h incubation, cells on the upper surface of the filters were removed with a cotton swab, and the filters were fixed with 100% methanol and stained with Giemsa dye. The cells that had invaded to the lower side of the filters were viewed under an Olympus microscope and counted in five fields of view. The invasive ability of the cancer cells was expressed as the mean number of cells in five fields.

### Western blot analysis

Cells were rinsed with ice-cold PBS and lysed in Triton ×-100 buffer (50 mmol/L HEPES, 150 mmol/L NaCl, 50 mmol/L NaF, 1% Triton ×-100, and 10% glycerol containing 5 mmol/L EDTA, 1 mmol/L phenylmethylsulfonyl fluoride, 10 μg/mL aprotinin, 10 μg/mL leupeptin, and 1 mmol/L sodium orthovanadate), and cell lysates were separated by SDS-PAGE and transferred to Immobilon membranes (Millipore Corp., MA), and followed by Western blot analysis as described previously [45].

### Cytotoxicity assays

Exponentially growing cell suspensions (5.0 × 10^3^ cells/100 μL) were seeded into 96-well plates. The following day, various concentrations of drugs were added. After incubation for 72 hr at 37°C, 20 μL of Cell Count Reagent SF (Nacalai Tesque) were added to each well and the plates were incubated for a further 1 to 2 hr at 37°C. Absorbance was measured at 450 nm with a 96-well plate reader. Triplicate wells were tested at each drug concentration. The IC_50_ value for each drug was calculated from the survival curves.

### Immunoprecipitation assay

Total protein from HAK-1A and HAK-1B cell lysates using lysis buffer was incubated for overnight with specifically antibody at 4°C, followed by incubation for 1 hour with Protein A/G plusagarose (Santa Cruz Biotechnology). The precipitates were washed thrice with ice-cold lysis buffer and resolved by SDS-PAGE followed by Western blot analysis. Anti-mTOR antibody for immunoprecipitation was purchased from Santa Cruz Biotechnology [45].

### Small interfering RNA transfection

HAK-1A and HAK-1B cells were transfected with siRNA using Lipofectamine RNAiMAX and Opti-MEM medium (Invitrogen) according to the manufacturer's recommendations. The siRNA corresponding to Rictor and control were purchased from Invitrogen. The siRNA corresponding to Raptor was purchased from QIAGEN.

### Statistical analysis

All results are expressed as mean ± SD of *n* observations, and statistical differences among the groups were assessed by two-tailed Student's *t*-test. A *P* value of less than 0.05 was considered significant.

## SUPPLEMENTARY MATERIALS


