# Supplementary material for: Phosphorylation of mTOR Ser2481 is a key target limiting the efficacy of rapalogs for treating hepatocellular carcinoma
Source: Oncotarget. 2016 Jun 18;7(30):47403–17. doi: 10.18632/oncotarget.10161 (PMC5216950; doi:10.18632/oncotarget.10161)
Supplement: Supplementary file 1 [file oncotarget-07-47403-s001.pdf]

Phosphorylation of mTOR Ser2481 is a key target limiting the efficacy of rapalogs for treating hepatocellular carcinoma

Supplementary Materials

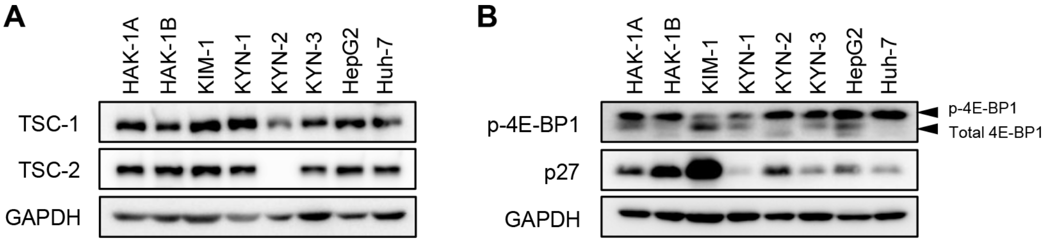

Supplementary Figure S1: Expression levels of mTORC1 signaling-related molecules in HCC cell lines. Western blot analysis of the expression in eight human HCC cell lines of TSC-1 and TSC-2 (**A**) and p-4E-BP1 and p27 (**B**).

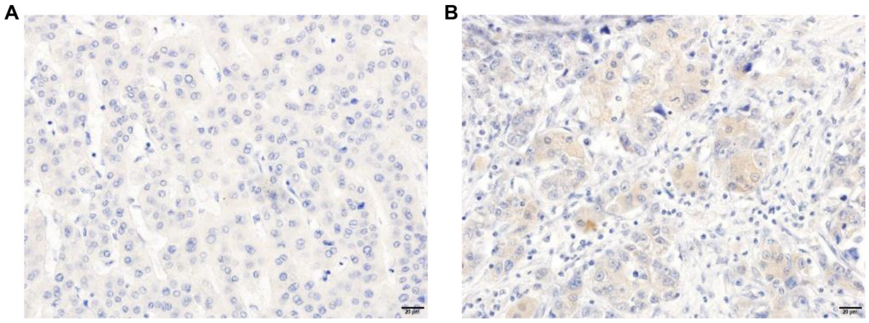

Supplementary Figure S2: Comparison of the expression level of keratin 19 between HAK-1A and HAK-1B. IHC analysis of the expression of keratin 19 in HAK-1A (**A**) and HAK-1B (**B**) tissues. Immunohistochemical stain was performed by keratin 19 antibody (BA17, DAKO, Glostrup, Denmark) using the streptavidin-biotin-peroxidase method.
